# L-Ascorbic Acid in the Epigenetic Regulation of Cancer Development and Stem Cell Reprogramming

**DOI:** 10.32607/actanaturae.11060

**Published:** 2020

**Authors:** A. P. Kovina, N. V. Petrova, S. V. Razin, O. L. Kantidze

**Affiliations:** Institute of Gene Biology Russian Academy of Sciences, Moscow, 119334 Russia

**Keywords:** vitamin C, cancer, stem cells, epigenome, chromatin

## Abstract

Recent studies have significantly expanded our understanding of the mechanisms of L-ascorbic acid (ASC, vitamin C) action, leading to the emergence of several hypotheses that validate the possibility of using ASC in clinical practice. ASC may be considered an epigenetic drug capable of reducing aberrant DNA and histone hypermethylation, which could be helpful in the treatment of some cancers and neurodegenerative diseases. The clinical potency of ASC is also associated with regenerative medicine; in particular with the production of iPSCs. The effect of ASC on somatic cell reprogramming is most convincingly explained by a combined enhancement of the activity of the enzymes involved in the active demethylation of DNA and histones. This review describes how ASC can affect the epigenetic status of a cell and how it can be used in anticancer therapy and stem cell reprogramming.

## INTRODUCTION

*L*-ascorbic acid (ASC, vitamin C) belongs to a class identified as essential water-soluble vitamins. Primates, guinea pigs, and fruit bats, compared to most mammals, have lost the ability to synthesize ASC due to a mutation in the gene of the *L*-gulonolactone oxidase (Gulo) that catalyzes the last stage of ASC synthesis from glucose [1]. The concentration of ASC in the human body is regulated by several mechanisms at once, which ensure a plasma ASC level of not more than 80 μM (oral intake) [2]. In this case, most mammalian cells maintain concentrations of intracellular ASC, which can reach 1–10 mM. The sodium-dependent transporters SVCT1 and 2 (*Figure*), which are differentially expressed in different tissues, are responsible for the active transport of ASC into cells [3].

**Fig. 1:**
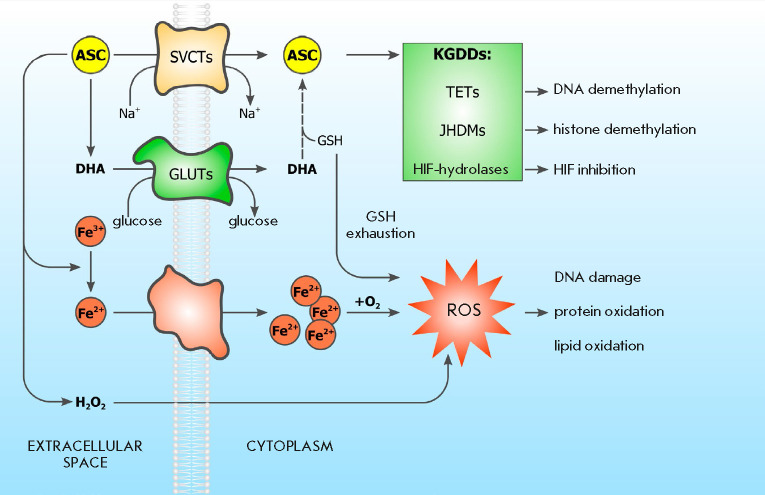
The role of ASC in the modulation of the epigenetic and redox statuses of the cell (see text for details). Abbreviations: ROS – reactive oxygen species; ASC – *L*-ascorbic acid; DHA – dehydroascorbic acid; GLUTs – glucose transporters; GSH – glutathione; HIFs – hypoxia-induced transcription factors; JHDM – JmjC-containing histone demethylases; KGDDs – α-ketoglutarate-dependent dioxygenases; SVCTs – Na^+^ and ASC transporters; TETs – methylcytosine dioxygenases

ASC is a good reducing agent: i.e., an electron donor. By donating the first electron, ASC transforms into the ascorbile radical, which is relatively stable and non-reactive. When it loses two electrons during two rounds of oxidation, ASC is converted to dehydroascorbic acid (DHA), which can be uptaken and released by the cell using the glucose transporters GLUT1, 2, 3, and 8 (*Figure*) [4]. Inside the cell, DHA can quickly get reduced to ASC by reaction with reduced glutathione (GSH) (*Figure*) [4]. In blood plasma, the reduced form of ASC predominates, while the DHA concentration is very low [5].

At micromolar concentrations, ASC can act as an antioxidant. ASC serves as a cofactor for several monooxygenases and Fe^2+^/α-ketoglutarate (α-KG)-dependent dioxygenases (KGDDs), acting as an electron donor (*Figure*) [6]. A classic example of α-KG-dependent dioxygenases is collagen-prolyl-4-hydroxylase (P4H), which has been well studied because decreased P4H activity causes scurvy. Accumulation of Fe3+ ions due to the activity of this enzyme leads to the inhibition of P4H activity and, therefore, to an incomplete hydroxylation of proline residues in the collagen molecule, aberrant collagen crosslinking, and scurvy symptoms [7]. ASC can reduce oxidized Fe3+ ions to catalytically active Fe^2+^ and, thus, prevent the development of scurvy. ASC, as a KGDD cofactor, affects important biological functions, such as catecholamine synthesis, collagen crosslinking, alkylated DNA repair, and hypoxia-inducible factor 1α (HIF-1α) degradation. A particular KGDD group consists of enzymes that catalyze hydroxylation of methylated nucleic acids (DNA and RNA) and methylated histones. Some of these dioxygenases require ASC as a cofactor in histone and DNA demethylation. The discovery of ASC-dependent KGDDs that are involved in the hydroxylation of methylated nucleic acid bases and histone amino acid residues suggests that ASC plays a role in the epigenetic regulation of gene expression.

## ASC AND DNA METHYLATION

Methylation of cytosine at the fifth position (5-methylcytosine (5mC)) is the most studied DNA modification occurring in mammals; it plays an important role in the epigenetic regulation of gene expression. Methylation of CpG nucleotides in promoters is usually associated with transcriptional repression and is involved in many processes, including X-chromosome inactivation and imprinting. 5mC is a very stable epigenetic label that can be removed in two ways: passive and active. Passive removal leads to dilution of the label during DNA replication in the absence of maintenance DNA methyltransferase (DNMT1) [8], while active demethylation is associated with the Ten-Eleven Translocation (TET) enzyme group that includes TET1–3 [9]. TETs are Fe^2+^/α-KG-dependent dioxygenases capable of sequential oxidation of 5mC to 5-hydroxymethylcytosine (5hmC), 5-formylcytosine (5fC), and 5-carboxylcytosine (5caC), which are recognized and removed by DNA repair enzymes [10, 11]. Unlike 5fC and 5caC, 5hmC is relatively stable; it can perform its own epigenetic function, because there exists a group of regulatory proteins that can specifically recognize and interact with 5hmC [10].

Because ASC is known to be a cofactor of some Fe^2+^/α-KG-dependent dioxygenases, ASC has been thought to be also a cofactor for TET-mediated DNA demethylation. Indeed, the addition of ASC to the culture medium was found to cause demethylation of several thousand genes in human embryonic stem cells (ESCs) [12]. In this regard, it is appropriate to recall that ASC promotes the formation of induced pluripotent stem cells (iPSCs) from terminally differentiated cells, which is accompanied by demethylation of the entire genome [13, 14]. *In vivo*, ASC was shown to enhance the generation of 5hmC in cultured cells. Most likely, ASC acts as a TET cofactor in the 5mC hydroxylation reaction [15, 16], because the addition of ASC dose-dependently increases the amount of 5hmC in mouse embryonic fibroblasts (MEFs) and this effect is abrogated by TET knockdown. The involvement of ASC in DNA demethylation has been observed in different cell types, as well as in model animals [17, 18, 19].

Interestingly, standard culture media lack ASC and the level of 5hmC in cultured cells is usually very low. Addition of ASC rapidly boosts the formation of 5hmC [20, 21]. This suggests that protein synthesis is not required for that task, but existing TET dioxygenases are activated [16]. According to the results of other experimental studies, ASC is required as a TET cofactor, but not just as a reducing agent. For example, addition of another reducing agent, GSH, did not change the 5hmC level; this indicates that the effect of ASC on 5hmC generation cannot be attributed to its role as a general reducing agent [16]. In mice with knockout of the *Gulo *gene (Gulo–/–), which is necessary for ASC biosynthesis, decreased amounts of 5hmC in various tissues were observed [19]. ASC was also shown to significantly increase the levels of all 5mC oxidation products, including 5fC and 5caC [17, 19]. ASC can also directly affect the functioning of TET family proteins, interacting with the C-terminal catalytic domain of enzymes, which probably promotes their correct folding and/or reuse of Fe^2+^ [19].

Therefore, there is convincing evidence that ASC acts as a cofactor of TET dioxygenases in 5mC oxidation, which is the first stage of active DNA demethylation.

## ASC AND HISTONE METHYLATION

Methylation of lysine and arginine residues in histones is an important epigenetic tool. While histone acetylation is usually considered an activating modification, methylation can be considered as a marker of both active (e.g., H3K4, H3K36, and H3K79) and inactive (e.g., H3K9, H3K27, and H4K20) chromatin [22]. Like DNA methylation, histone methylation was initially considered an irreversible post-translational modification. In the early 2000s, the lysine-specific histone demethylases KDM1A (LSD1) and KDM1B (LSD2) were discovered. They are capable of demethylating only mono- and di-, but not trimethylated lysine residues in the histone molecule [23, 24]. Later, the enzyme KDM4A (JHDM3A) was discovered, which is also capable of removing the third methyl group from the lysine residues 9 and 36 in the histone H3 molecule [25]. Further, other similar enzymes were identified, which, like KDM4A, contain the Jumonji C (JmjC) domain. This catalytic domain provides the hydroxylase activity of demethylases, which is necessary for the demethylation of the amino acid residues in histones [26]. JmjC domain-containing demethylases also belong to the family of Fe^2+^/α-KG-dependent dioxygenases, the general functioning principles and cofactors of which were discussed earlier [25, 27].

JmjC-containing enzymes were found to require ASC. *In vitro*, ASC is required for both KDM2A and KDM3A (JHMD2A): the activity of these enzymes was in correlation with the amount of ASC in the reaction buffer [27]; in this case, KDM4A completely lost its catalytic activity in *in vitro *experiments upon removal of ASC from the medium [25].

The investigation of the differentiation of various cells has demonstrated that this process is significantly impaired in the absence of ASC due to the inability of cells to control the repressive histone modification level. For example, the absence of ASC during the endothelial-to-hematopoietic transition leads to an accumulation of H3K27me3 in the genomic loci that are important for hematopoiesis [28]. An excess of ASC underlies the loss of histone H3 lysine 9 dimethylation (H3K9me2) within extended genomic domains in mouse embryonic stem cells (LOCK domains [29]), which is apparently caused by the stimulation of the demethylases Kdm3a and Kdm3b [30]. Addition of ASC to T-lymphocytes leads to a decrease in the H3K9me3 level in the *cis*-regulatory elements of the interleukin-17 (IL-17) gene locus, due to the activation of histone demethylase KDM4A and, accordingly, to an increase in the IL-17 expression [31]. In addition, ASC was shown to stimulate histone demethylation during both the initial stages of reprogramming of somatic cells into iPSCs [32] and the transition from pre-iPSCs to completely reprogrammed iPSCs [33, 34]. All these findings suggest that ASC is a cofactor of JmjC-containing histone demethylases and that it modulates histone demethylation, most likely through the regeneration of the catalytically active Fe^2+^.

## ASC AND CANCER

Any low-molecular-weight compounds capable of modifying epigenetic profiles are considered potential anticancer agents. The question of whether ASC may be used as an anticancer agent has been debated for decades. Interest in the possible use of ASC in cancer therapy emerged back in the 1970s, when Pauling and Cameron reported an increased survival rate in patients with late-stage cancer after an intravenous administration of ASC (10 g per day), but later attempts to repeat these results failed [35]. This was related to the method used for ASC delivery: later studies used oral administration, which prevented the achieving of therapeutically significantly high ASC concentrations in the blood [36]. Further research led to the emergence of new hypotheses about the potential mechanisms underlying the anticancer activity of ASC. As in the case of other chemotherapeutic agents, different tumor types exhibit different sensitivities to the cytotoxic effect of ASC [37]. ASC concentrations of about 2–5 mM are sufficient to reduce the survival rate of most *in vitro *cultured cancer cells by 50%. At the same time, many non-cancerous cells maintain normal activity at ASC concentrations of about 20 mM [37]. It should, however, be noted that about 10–15% of cancer cell types are insensitive to ASC even at a concentration of 20 mM.

**Potential mechanisms of anticancer activity of ASC**

The mechanisms of anticancer activity of ASC can be divided into two groups: mechanisms affecting redox biology, and mechanisms associated with the function of ASC as a cofactor of α-KG-dependent dioxygenases (*Figure*).

The first group includes two mechanisms that are not mutually exclusive, and their combined action may result in ASC toxicity to cancer cells. The prooxidant properties of ASC at millimolar (pharmacological) concentrations may increase the amount of non-reparable lesions to a cancer cell. ASC accelerates the Fe^2+^-dependent production of the hydroxyl radical (•OH) from H_2_O_2_ through oxidation of Fe3+ ions to labile iron ions (Fe^2+^), thereby continuously generating reactive oxygen species (ROS) and promoting cell death [38]. In addition, spontaneous autooxidation of ASC by oxygen can lead to the accumulation of H_2_O_2_, high concentrations of which cause cell death (*Figure*) [37, 39, 40].

The second mechanism from this group is extracellular oxidation of ASC to DHA that is structurally similar to glucose and is transported into cells via GLUT transporters, which promotes an increase in the intracellular DHA pool. Cancer cells can transport DHA into the cell, where it is reduced to ASC, which leads to the depletion of the pool of glutathione and NADH-and NADPH-dependent enzymes [4]. This, in turn, causes oxidative stress and inactivation of glyceraldehyde-3-phosphate dehydrogenase, inhibits glycolysis, the level of which is increased in cancer cells, and leads to an energy crisis that is fatal to cells (*Figure*) [41, 42].

As a cofactor of Fe^2+^/α-KG-dependent dioxygenases, ASC can also significantly affect the viability of cancer cells. Hypoxia-induced transcription factors (HIFs) increase the expression of the genes responsible for a successful adaptation of cancer cells to the hypoxia caused by rapid cell division and insufficient vascularization of a growing tumor [43]. HIF activity is controlled by HIF hydroxylases that modify, at normal conditions (normoxia), subunits of these factors, which promotes their proteasomal degradation [44]. HIF-hydroxylases belong to the family of dioxygenases, and ASC may be their cofactor [45]. ASC-deficient cells exhibit reduced HIF-hydroxylase activity and, therefore, an increased level of HIF-factor transcription, in particular in mild or moderate hypoxia [46-48]. These findings suggest that the addition of ASC to cancer cells may stimulate the activity of HIF hydroxylases and decrease HIF activity, thereby slowing down the rate of tumor growth (*Figure*) [49, 50].

As a cofactor of the enzymes of the Fe^2+^/α-KG-dependent dioxygenase family, ASC influences the epigenetic alterations that are often inextricably linked with the development of cancer (*Figure*). There are important epigenetic alterations characteristic of cancers. First, one of the cancer markers is the global DNA hypomethylation that can activate a transcription of transposons and oncogenes, which leads to changes in gene expression and, subsequently, to carcinogenesis [51]. Second, it is the hypermethylation of tumor suppressor gene promoters. As was recently shown, the hydroxymethylation level (5hmC) can also change in some cancers [10]. The possibilities of using ASC to modulate the epigenetic status of cancer cells are discussed in detail in the next section.

**Biomarkers for using ASC in anticancer therapy**

In recent years, growing interest has been directed at the role of ASC in the modulation of DNA and histone methylation profiles, which is due to the fact that ASC is a cofactor of the enzymes involved in the demethylation of DNA (TET) and histones (JmjC-containing demethylases) [9, 52]. Changed expression levels of these enzymes and/or mutations in their genes have been found both in various solid tumors and in hematological malignancies. Because mutations usually involve only one copy of the gene, the addition of ASC can compensate for the effect of this mutation through an increased activity of the remaining non-mutant enzyme [52].

Mutations in the *TET *genes are observed in hematological malignancies, both myeloid and lymphoid [53], and usually lead to DNA hypermethylation [54-56]. In this case, ASC acts as an epigenetic modulator: in ASC-treated cancer cells, the TET activity is increased, which leads to DNA demethylation, and expression of tumor suppressor genes, such as Smad1, is increased [55].

Mutations in cancers often involve genes that are directly associated with the TET activity. For example, the isocitrate dehydrogenases IDH1 and IDH2, which are required for the production of the TET cofactor α-KG, are often mutated in hematological malignancies, as well as in some subtypes of gliomas and solid tumors [57]. In most cases, these mutations lead to an increased level of 2-hydroxyglutarate and, as a consequence, to DNA hypermethylation and reduced 5hmC levels. Several studies have been performed on mouse and cell models of leukemia caused by mutations in the *TET2 *or *IDH1 *gene [52, 55, 58, 59]. Upon intravenous administration of ASC, as well as upon restoration of *TET2 *expression, DNA hypermethylation was suppressed or decreased due to increased DNA demethylation [52, 55, 59]. Interestingly, after the addition of ASC, leukemia cells became more sensitive to the inhibition of poly(ADP-ribose) polymerases (PARPs), which can be used as an effective, combined strategy for the treatment of cancers with mutations in the *TET *gene [52]. The effect of ASC addition was also tested on IDH1 mutant mouse leukemia cells [59]. ASC was shown to induce a TET2-dependent increase in the amount of 5hmC, loss of 5mC, and increased expression, which was in correlation with a decreased self-renewal of leukemic stem cells and enhanced differentiation towards the mature myeloid phenotype [59]. These data indicate that ASC can, at least in part, mitigate the effect of TET and IDH loss.

Brain tissues possess the highest need in intracellular ASC, because it is involved in the enhancement of the biosynthesis of norepinephrine and acts as a cofactor of dopamine-β-hydroxylase, as well as an inhibitor of glutamate uptake in retinal neurons. An oxidized form of ASC (DHA) is able to penetrate the blood-brain barrier and then accumulate in the stem cells of the cortex and cerebellum, the neurons, and neuroblastoma cells [60, 61]. The mechanism of ASC action in glioma is believed to have something to do with its prooxidant properties. Clinical studies have shown that the combination of conventional therapies with intravenous administration of high ASC doses improves the quality of life of glioblastoma patients, increases their overall survival likelihood, and arrests the progression of the disease [62, 63].

The genes of fumarate hydratase (FH) and succinate dehydrogenase (SDH) are mutated in many cancer types [64, 65]. Mutations in these genes lead to the accumulation of succinate and fumarate, which act as oncometabolites, competitively inhibiting TET and JmjC-containing histone demethylases, even in the presence of stable α-KG levels [66]. Indeed, *FH *or *SDH *knockdown in mouse liver cells has led to a decrease in the 5hmC level [66]. The effect of ASC on cells with mutations in the *FH *or *SDH *gene has not yet been explored, but it may be suggested that enhancement of the enzymatic activity of TET or JmjC-containing demethylases may be sufficient to restore the normal epigenetic landscape, even in the presence of inhibitory oncometabolites.

**ASC as adjuvant therapy**

Potential interactions between ASC and chemotherapeutic agents have long been a controversial issue [67]. Animal studies have shown that the simultaneous use of high ASC doses and various chemotherapeutic agents slows the growth of a xenograft tumor [68, 69, 70]. Many *in vivo *studies have shown that orally or intravenously administered ASC decreases the level of general toxicity of chemotherapeutic agents [71]. ASC administration reduced leukocyte loss, weight loss, accumulation of ascites, hepatotoxicity, lipid oxidation, and chemotherapy-induced cardiomyopathy in [69, 72].

In clinical trials involving patients with different types of cancer, intravenous administration of high ASC doses, together with chemotherapeutic agents, showed no side effects and, in many cases, improved health and quality of life [69, 73, 74]. It has been often noted that combination therapy involving ASC increases sensitivity to certain anticancer drugs and, therefore, has the potential to reduce the required dose and side effects [52, 75]. A reduction in chemotherapy-associated toxicity was observed, e.g., in patients with stage III–IV ovarian cancer who had received carboplatin and paclitaxel, in combination with a high dose of ASC [69].

The large-scale DNA demethylation observed upon the addition of ASC to human leukemia cell lines is associated with the increased TET2 activity in them [52, 76]. DNA methyltransferase inhibitors (DNMTis) such as 5-azacytidine and decitabine reduce aberrant DNA hypermethylation by suppressing the activity of supporting and *de novo *DNA methyltransferases [77]. The synergistic action of ASC and DNMTi causes both passive and active DNA demethylation, which leads to cancer cell proliferation inhibition and apoptosis [76]. The results of clinical trials performed to date confirm, in general, the efficacy of a combined use of ASC and DNMTi [74].

ASC enhances the cytotoxic effect of a PARP1/2 inhibitor, olaparib, on human acute myeloid leukemia (AML) cells [52]. Probably, this is a case of synthetic lethality: TET-mediated DNA oxidation caused by ASC sensitizes AML cells to PARP inhibition due to the impossibility of removing non-canonical bases from DNA.

ASC also increases the sensitivity of melanoma cells to the bromodomain and extraterminal motif-containing protein inhibitors (BETi) that cause changes in the level of histone acetylation and are considered promising agents for the treatment of cancers [75]. ASC enhances the effectiveness of BETi by decreasing the level of histone H4 acetylation via the TET-dependent suppression of the histone acetyltransferase 1 (HAT1) expression.

In the population, the average rate of ASC deficiency is low, but it is much higher in patients with advanced cancer [78]. ASC deficiency is detected in most patients with hematological malignancies [76, 79]. Even in the absence of mutations in the *TET *genes, ASC deficiency can further impair the function of TET proteins upon suppression of tumor progression. Administration of some anticancer drugs, such as cisplatin, fluorouracil, nilotinib, and interleukin-2, was shown to significantly reduce the ASC level [80, 81]. Therefore, ASC deficiency can increase the aggressiveness of the disease and increase the risk of a relapse.

## ASC AND STEM CELL REPROGRAMMING

**ASC and embryonic development**

In the early stages of mammalian embryonic development, there are two rounds of DNA demethylation that occurs in both passive and active ways. Immediately after fertilization, 5mC in the paternal chromatin is quickly replaced by 5hmC via TET3-mediated hydroxylation, after which the formed 5hmC is diluted during the DNA replication of implanted embryos [82]. This leads to an almost complete disappearance of the 5mC pattern in the paternal chromatin as early as at the stage of 16 cells – methylation is retained only at imprinted genomic loci [82, 83]. Maternal chromatin demethylation, which occurs a little later, is also mediated by both TET3-dependent oxidation and passive demethylation [84, 85]. After embryo implantation, the internal cell mass, which gives rise to the embryo, undergoes *de novo *DNA methylation [86]. The second stage of DNA demethylation, which includes, inter alia, demethylation of imprinted loci, occurs in primary germ cells [87, 88].

A significant amount of ASC, as a cofactor, is required to satisfy the cell’s TET needs, and the lack of ASC can impair embryonic development due to incomplete DNA demethylation, which may lead to congenital anomalies. ASC is required for TET-dependent demethylation of many promoters and activation of germline genes in mouse and human embryonic stem cells [12, 17]. Histone demethylation mediated by JmjC-containing histone demethylases is critical for embryonic development [89-92]. Maternal and paternal nutrition was shown to affect DNA and histone methylation patterns in offspring cells [93, 94]. As shown in a mouse model, ASC consumption is necessary for proper DNA demethylation and further development of female germ cells in the fetus [95]. ASC deficiency in the mother does not affect the overall development of the fetus, but it leads to a decreased amount of germ cells, delayed meiosis, and reduced fertility in offspring [95]. The effects of ASC deficiency in pregnancy are partially similar to those of *TET1 *knockout.

In general, ASC, supporting the catalytic activity of TET and some JmjC-containing histone demethylases, especially during epigenetic reprogramming, may be required in the early stages of embryonic development.

**ASC and somatic cell reprogramming**

The ability to reprogram somatic cells into iPSCs that can further be used to produce various differentiated cell populations is an important tool in regenerative medicine [96, 97]. Induction of the transcription factors Oct4, Sox2, Klf4, and c-Myc (OSKM) leads to the production of iPSCs from differentiated somatic cells [96, 98, 99]. The effectiveness of the reprogramming is low due to factors such as the age of the cell donor, number of passages in the culture, and the tissue origin of the cells [100, 101, 102]. Reprogramming is based on two main processes: repression of differentiation genes and activation of the genes that regulate pluripotency. Removal of epigenetic modifications in the genome of somatic cells is critical to the success of reprogramming [103]. Numerous studies in the past decade have shown that the addition of ASC to a medium of cultured somatic cells increases the effectiveness of reprogramming and the quality of the obtained iPSCs [13, 14, 34]. By enhancing the catalytic activity of TET and JmjC-containing histone demethylases, ASC stimulates histone and DNA demethylation in somatic cells, which may simultaneously activate the expression of pluripotency genes and erase the epigenetic memory of the differentiated state in mature cells.

In the first studies, ASC was added to the culture medium for reprogramming as an antioxidant to mitigate the effects of ROS, the level of which was increased upon induced expression of OSKM [104]. However, ASC enhanced the proliferation of ESCs and generation of iPSCs from mouse and human fibroblasts more efficiently than other antioxidants [13]. ASC is supposed to promote cell reprogramming because of the increased histone demethylation that is necessary for the expression of Nanog, one of the main transcription factors [105]. Indeed, addition of ASC-dependent KGDD inhibitors impaired iPSC formation from MEFs [34].

One of the obstacles to somatic cell reprogramming is histone H3K9 methylation [33]. Addition of ASC to the pre-iPSCs occurring in an intermediate reprogramming state leads to their transformation into fully reprogrammed iPSCs [13]. This may be explained by the fact that the presence of ASC promotes a more efficient demethylation of histone H3K9 associated with the genes of pluripotency-regulating transcription factors, which leads to an increase in their expression [33]. The effectiveness of reprogramming increases upon simultaneous addition of ASC and inhibition of H3K9-specific methyltransferases [13]. Genome-wide screening using RNA interference helped to identify histone demethylase Kdm3b (Jhdm2b) as the main target activated by ASC during cell reprogramming [33]. Also, an increase in the activity of the demethylases Kdm3a/b (Jmjd1a/b) and Kdm4b/c (Jmjd2b/c) by ASC in mouse ESCs and in pre-iPSCs was shown to lead to a specific loss of H3K9me2/me3 in the loci of the genes responsible for pluripotency [30, 33].

Another JmjC-containing enzyme from the Kdm group, Kdm6a (Utx), demethylates H3K27me3 and is the most important regulator of pluripotency induction during the reprogramming of mouse and human somatic cells [106]. Addition of ASC to the culture medium of mouse ESCs alters the distribution of H3K27me3 in their genome, and this occurs mainly locus-specifically [30], the reasons for which remain to be clarified.

An analysis of changes in the methylated H3K36 profiles during the reprogramming of MEFs into iPSCs demonstrated that ASC causes a noticeable decrease in H3K36me2/3 due to an increase in the activity of the histone demethylases Kdm2a/2b (Jhdm1a/1b) [34]. This, inter alia, decreases the expression level of cyclin-dependent kinase inhibitor genes at the INK4/ARF locus and removes restrictions on the reprogramming of somatic cells [101, 107]. Reprogramming using expression of Oct4 and histone demethylase KDM2B in the presence of ASC is known to activate the expression of the miR302/367 microRNA cluster [34]. KDM2B causes an ASC-dependent decrease in the methylation levels of H3K36 that surrounds the Oct4 binding sites located near the miR302/367 gene and promotes their expression [34]. The miR302/367 cluster regulates pluripotency by inhibiting the expression of the genes important for differentiation [108]. Because these microRNAs play a decisive role in maintaining cell pluripotency, their expression decreases during differentiation [109]. It is noteworthy that expression of the entire miR302/367 cluster is sufficient for the reprogramming of fibroblasts [110].

Expression of *TET *genes plays an important role in somatic cell reprogramming. Knockdown of *TET *genes significantly complicates, and in some cases even completely prevents, the reprogramming of MEFs into iPSCs by the expression of OSKM [20, 111, 112]. As expected, ASC increases the effectiveness of reprogramming mouse and human fibroblasts into iPSCs in a TET-dependent manner [16-19]. For a more efficient reprogramming of mouse iPSCs into the naive pluripotency state, ASC can be used, together with vitamin A (retinoic acid), which activates TET2 and TET3 transcription through specific signaling pathways [13, 113, 114].

Along with its important role in somatic cell reprogramming, ASC is also required in order to maintain proliferation and a normal differentiation potential for ESCs, iPSCs, neuronal stem cells, and mesenchymal stem cells [115]. Most likely, the involvement of ASC in the prevention of premature aging for these cell cultures and the preservation of their epigenetic plasticity is mediated by its role as a cofactor of DNA and histone demethylation enzymes.

## CONCLUSION

Recent studies have significantly expanded our understanding of the mechanisms underlying ASC action, which has produced several hypotheses that validate the possibility of its use in clinical practice. ASC may be considered an epigenetic drug capable of reducing aberrant DNA and histone hypermethylation, which may be helpful in the treatment of some cancers and neurodegenerative diseases. A correct understanding of the mechanisms of ASC action and the ongoing clinical studies will help identify the types of cancer patients that may benefit from a high-dose ASC treatment. Intravenous administration of ASC can act alone, or in combination with different chemotherapeutic agents. Preclinical and clinical trials have demonstrated that the toxicity and side effects of chemotherapy in this case can be mitigated without decreasing tumor-specific cytotoxic activity. On the other hand, the clinical significance of ASC is associated with regenerative medicine, in particular with the production of iPSCs from somatic cells. The effect of ASC on somatic cell reprogramming is most convincingly explained by the combined enhancement of the activity of the enzymes involved in the active demethylation of DNA and histones.

## Abbreviations

5hmC: 5-hydroxymethylcytosine
5mC: 5-methylcytosine
α-KG: α-ketoglutarate
AML: acute myeloid leukemia
ASC: L-ascorbic acid
BETi: bromodomain and extraterminal inhibitors
DHA: dehydroascorbic acid
DNMT: DNA methyltransferase
DNMTi: DNMT inhibitors
GSH: glutathione
Gulo: L-gulonolactone oxidase
IDH: isocitrate dehydrogenase
KGDD: α-KG-dependent dioxygenase
MEFs: mouse embryonic fibroblasts
P4H: prolyl 4-hydroxylase
PARP: poly(ADP-ribose)polymerase
TET: ten-eleven translocation dioxygenase
iPSCs: induced pluripotent stem cells
